# 4-Bromo­seleno­anisole

**DOI:** 10.1107/S160053680902296X

**Published:** 2009-06-24

**Authors:** Henning Osholm Sørensen, Nicolai Stuhr-Hansen

**Affiliations:** a Center for Fundamental Research: Metal Structures in Four Dimensions, Risø National Laboratory for Sustainable Energy, Technical University of Denmark, Frederiksborgvej 399, P.O. 49, DK-4000 Roskilde, Denmark; b Department of Medicinal Chemistry, University of Copenhagen, Universitetsparken 2, DK-2100 Copenhagen, Denmark

## Abstract

The title compound, 1-bromo-4-methyl­seleno­benzene, C_7_H_7_BrSe, was prepared by methyl­ation of 4-bromo­seleno­phenolate with methyl iodide, and crystals suitable for structure determination were obtained by sublimation. The mol­ecule is essentially planar; the Se—Me bond is rotated by only 2.59 (19)° out of the least-squares plane of the benzene ring. The most pronounced intermolecular interactions are two hydrogen bonds of the type C—H⋯π, which determine a herring-bone pattern in the crystal packing.

## Related literature

For related selenobenzene structures, see: Oddershede *et al.* (2003 ▶); Sørensen & Stuhr-Hansen (2009 ▶); Stuhr-Hansen *et al.* (2009 ▶). For the ^77^Se-NMR spctrum, see: Eggert *et al.* (1986 ▶). For the melting point, see: Gilow *et al.* (1968 ▶).
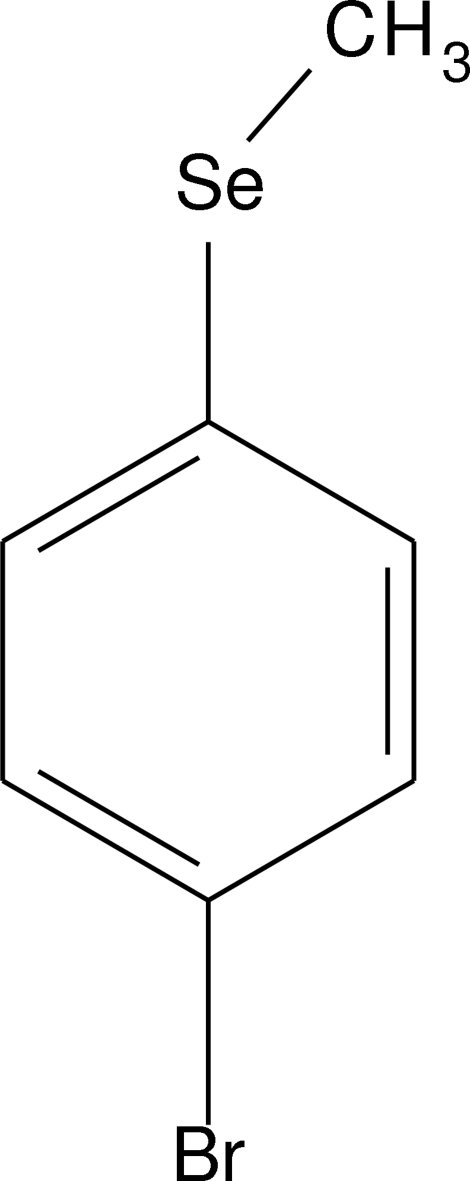


## Experimental

### 

#### Crystal data

C_7_H_7_BrSe
*M* *_r_* = 250.00
Orthorhombic, 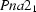

*a* = 5.8298 (8) Å
*b* = 7.0671 (11) Å
*c* = 18.776 (6) Å
*V* = 773.6 (3) Å^3^
*Z* = 4
Cu *K*α radiation
μ = 11.86 mm^−1^
*T* = 122 K
0.36 × 0.09 × 0.09 mm

#### Data collection

Enraf–Nonius CAD-4 diffractometer
Absorption correction: numerical (DeTitta, 1985 ▶) *T*_min_ = 0.145, *T*_max_ = 0.454
5823 measured reflections
1590 independent reflections
1590 reflections with *I* > 2σ(*I*)
*R*_int_ = 0.031
5 standard reflections frequency: 166.7 min intensity decay: 8.7%

#### Refinement

*R*[*F* ^2^ > 2σ(*F* ^2^)] = 0.027
*wR*(*F* ^2^) = 0.075
*S* = 1.15
1590 reflections
83 parameters
1 restraint
H-atom parameters constrained
Δρ_max_ = 0.62 e Å^−3^
Δρ_min_ = −1.30 e Å^−3^
Absolute structure: Flack (1983 ▶)
Flack parameter: −0.01 (4)

### 

Data collection: *CAD-4 EXPRESS* (Enraf–Nonius, 1994 ▶); cell refinement: *CAD-4 EXPRESS*; data reduction: *DREAR* (Blessing, 1987 ▶); program(s) used to solve structure: *SHELXS97* (Sheldrick, 2008 ▶); program(s) used to refine structure: *SHELXL97* (Sheldrick, 2008 ▶); molecular graphics: *ORTEPII* (Johnson, 1976 ▶) and *PLATON* (Spek, 2009 ▶); software used to prepare material for publication: *SHELXL97*.

## Supplementary Material

Crystal structure: contains datablocks I, global. DOI: 10.1107/S160053680902296X/fj2225sup1.cif

Structure factors: contains datablocks I. DOI: 10.1107/S160053680902296X/fj2225Isup2.hkl

Additional supplementary materials:  crystallographic information; 3D view; checkCIF report

## Figures and Tables

**Table 1:** Selected bond lengths (Å)

Se1—C1	1.916 (4)
Se1—C7	1.930 (5)
Br1—C4	1.906 (4)

**Table 2:** Hydrogen-bond geometry (Å, °)

*D*—H⋯*A*	*D*—H	H⋯*A*	*D*⋯*A*	*D*—H⋯*A*
C2—H2⋯C2^i^	0.95	2.84	3.747 (4)	159
C5—H5⋯C5^ii^	0.95	2.83	3.740 (5)	160

Symmetry codes: (i) 
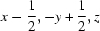
; (ii) 
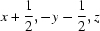
.

**Fig. 1.**
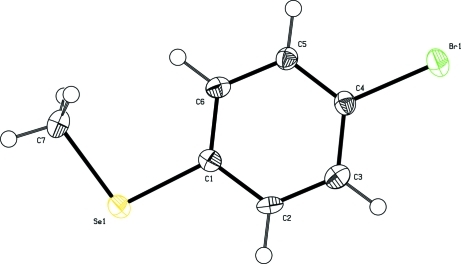
Ortep drawing (Johnson, 1976) of the title compound including labelling of the atoms. The displacement ellipsoids are drawn at the 50% probability level. H atoms are shown as spheres with an arbitrary radii.

## supplementary crystallographic information

### Experimental

The title compound was synthesized as described below. To a stirred solution containing di(4-bromophenyl) diselenide (2.35 g, 5 mmol) and hydrazine hydrate (2.75 mmol) in DMSO (8 ml) was added 25% methanolic sodium methanolate (approximately 2 g, the last 0.2 g added dropwise with intervals of 5 s until the yellow color of di(4-bromophenyl) diselenide disappeared). 4-Methyliodide (1.70 g, 12 mmol) was added and the reaction mixture was further stirred for 10 minutes. The clear colourless reaction mixture was diluted with water (100 ml) and extracted with ether (3 *x* 25 ml). The combined organic phases were washed with water (15 ml), filtered through alumina (neutral, 6 g) by means of pentane and the solvent was evaporated *in vacuo*. Sublimation (200 °C, 5 m mH g) gave the title compound 4-bromoselenoanisol (2.24 g, 90%) as long white needles in a quality suitable for structure determination by single-crystal *x*-ray diffraction; mp 47–48 °C (lit. (Gilow *et al.*, 1968) mp 46–47 °C). C_7_H_7_BrSe: found C 33.69% H 2.57%; calc. C 33.63% H 2.82%. Mass spectrum (EI; m/*z*, relative intensity): 250 (*M*^+^, 100), 235 (67), 171 (7), 156 (56). ^1^H-NMR (CDCl_3_) δ: 2.33 (3*H*, s), 7.26 (2*H*, d, J = 8.6 Hz), 7.36 (2*H*, d, J = 8.6 Hz). 77Se-NMR (Eggert *et al.*, 1986) (CDCl_3_) δ: 211 p.p.m..

### Refinement

Hydrogen atoms were found in the difference Fourier map. All hydrogen atoms were treated as riding atoms with C—H distances of 0.95 for C_ar_ and 0.98 for the C_Me_. Isotropic displacement parameters for all H atoms were constrained to 1.2*U*_eq_ of the connected non-hydrogen atom (1.5*U*_eq_ for Me groups).

### Crystal data

**Table:**

C_7_H_7_BrSe	*F*(000) = 472
*M**_r_* = 250.00	*D*_x_ = 2.147 Mg m^−^^3^
Orthorhombic, *P**n**a*2_1_	Cu *K*α radiation, λ = 1.54184 Å
Hall symbol: P 2c -2n	Cell parameters from 20 reflections
*a* = 5.8298 (8) Å	θ = 39.2–40.3°
*b* = 7.0671 (11) Å	µ = 11.86 mm^−^^1^
*c* = 18.776 (6) Å	*T* = 122 K
*V* = 773.6 (3) Å^3^	Needle, white
*Z* = 4	0.36 × 0.09 × 0.09 mm

### Data collection

**Table:**

Enraf–Nonius CAD-4 diffractometer	1590 reflections with *I* > 2σ(*I*)
Radiation source: fine-focus sealed tube	*R*_int_ = 0.031
graphite	θ_max_ = 74.8°, θ_min_ = 4.7°
ω–2θ scans	*h* = −7→7
Absorption correction: numerical (DeTitta, 1985)	*k* = −8→8
*T*_min_ = 0.145, *T*_max_ = 0.454	*l* = −23→23
5823 measured reflections	5 standard reflections every 166.7 min
1590 independent reflections	intensity decay: 8.7%

### Refinement

**Table:**

Refinement on *F*^2^	Hydrogen site location: inferred from neighbouring sites
Least-squares matrix: full	H-atom parameters constrained
*R*[*F*^2^ > 2σ(*F*^2^)] = 0.027	*w* = 1/[σ^2^(*F*_o_^2^) + (0.0537*P*)^2^ + 0.4964*P*] where *P* = (*F*_o_^2^ + 2*F*_c_^2^)/3
*wR*(*F*^2^) = 0.075	(Δ/σ)_max_ < 0.001
*S* = 1.15	Δρ_max_ = 0.62 e Å^−^^3^
1590 reflections	Δρ_min_ = −1.29 e Å^−^^3^
83 parameters	Extinction correction: *SHELXL97* (Sheldrick, 2008), Fc^*^=kFc[1+0.001xFc^2^λ^3^/sin(2θ)]^-1/4^
1 restraint	Extinction coefficient: 0.0128 (5)
Primary atom site location: heavy-atom method	Absolute structure: Flack (1983)
Secondary atom site location: difference Fourier map	Flack parameter: −0.01 (4)

### Special details

**Table:**

Geometry. All e.s.d.'s (except the e.s.d. in the dihedral angle between two l.s. planes) are estimated using the full covariance matrix. The cell e.s.d.'s are taken into account individually in the estimation of e.s.d.'s in distances, angles and torsion angles; correlations between e.s.d.'s in cell parameters are only used when they are defined by crystal symmetry. An approximate (isotropic) treatment of cell e.s.d.'s is used for estimating e.s.d.'s involving l.s. planes.
Refinement. Refinement of *F*^2^ against ALL reflections. The weighted *R*-factor *wR* and goodness of fit *S* are based on *F*^2^, conventional *R*-factors *R* are based on *F*, with *F* set to zero for negative *F*^2^. The threshold expression of *F*^2^ > σ(*F*^2^) is used only for calculating *R*-factors(gt) *etc*. and is not relevant to the choice of reflections for refinement. *R*-factors based on *F*^2^ are statistically about twice as large as those based on *F*, and *R*- factors based on ALL data will be even larger.

### Fractional atomic coordinates and isotropic or equivalent isotropic displacement parameters (Å^2^)

**Table:**

	*x*	*y*	*z*	*U*_iso_*/*U*_eq_	
Se1	−0.08215 (7)	0.05711 (4)	0.00008 (2)	0.02110 (15)	
Br1	0.38155 (8)	−0.04502 (5)	0.317143 (19)	0.02756 (16)	
C1	0.0636 (7)	0.0211 (5)	0.0905 (2)	0.0176 (7)	
C2	−0.0585 (6)	0.0834 (5)	0.1507 (2)	0.0185 (7)	
H2	−0.2055	0.1395	0.1450	0.022*	
C3	0.0336 (8)	0.0636 (4)	0.2178 (2)	0.0219 (8)	
H3	−0.0478	0.1066	0.2586	0.026*	
C4	0.2483 (6)	−0.0207 (5)	0.22490 (18)	0.0184 (7)	
C5	0.3691 (6)	−0.0843 (5)	0.1666 (2)	0.0184 (7)	
H5	0.5149	−0.1420	0.1727	0.022*	
C6	0.2759 (7)	−0.0633 (4)	0.0982 (2)	0.0193 (8)	
H6	0.3579	−0.1067	0.0577	0.023*	
C7	0.1556 (9)	−0.0397 (6)	−0.0615 (3)	0.0300 (9)	
H7A	0.2997	0.0268	−0.0517	0.045*	
H7B	0.1117	−0.0195	−0.1113	0.045*	
H7C	0.1759	−0.1753	−0.0528	0.045*	

### Atomic displacement parameters (Å^2^)

**Table:**

	*U*^11^	*U*^22^	*U*^33^	*U*^12^	*U*^13^	*U*^23^
Se1	0.0191 (2)	0.0252 (2)	0.0190 (2)	0.00090 (10)	−0.00294 (17)	0.00115 (15)
Br1	0.0276 (2)	0.0363 (3)	0.0188 (2)	0.00065 (13)	−0.00506 (19)	0.00065 (16)
C1	0.0174 (18)	0.0161 (14)	0.0193 (18)	−0.0014 (12)	−0.0005 (13)	0.0019 (13)
C2	0.0120 (15)	0.0178 (15)	0.026 (2)	0.0030 (12)	0.0026 (13)	−0.0003 (13)
C3	0.022 (2)	0.0201 (16)	0.024 (2)	0.0001 (11)	0.0034 (17)	−0.0019 (12)
C4	0.0186 (19)	0.0202 (16)	0.0166 (17)	−0.0032 (12)	−0.0007 (15)	0.0015 (12)
C5	0.0168 (15)	0.0206 (14)	0.0177 (18)	−0.0018 (12)	0.0009 (13)	−0.0014 (13)
C6	0.0170 (19)	0.0178 (16)	0.0230 (19)	0.0009 (10)	0.0012 (16)	−0.0022 (11)
C7	0.032 (2)	0.039 (2)	0.019 (2)	0.0061 (15)	0.0035 (18)	−0.0038 (14)

### Geometric parameters (Å, °)

**Table:**

Se1—C1	1.916 (4)	C3—H3	0.9500
Se1—C7	1.930 (5)	C4—C5	1.377 (5)
Br1—C4	1.906 (4)	C5—C6	1.401 (6)
C1—C6	1.382 (5)	C5—H5	0.9500
C1—C2	1.406 (5)	C6—H6	0.9500
C2—C3	1.377 (6)	C7—H7A	0.9800
C2—H2	0.9500	C7—H7B	0.9800
C3—C4	1.392 (6)	C7—H7C	0.9800
			
C1—Se1—C7	99.5 (2)	C4—C5—C6	119.7 (3)
C6—C1—C2	120.3 (4)	C4—C5—H5	120.2
C6—C1—Se1	123.2 (3)	C6—C5—H5	120.2
C2—C1—Se1	116.5 (3)	C1—C6—C5	119.3 (4)
C3—C2—C1	120.4 (4)	C1—C6—H6	120.4
C3—C2—H2	119.8	C5—C6—H6	120.4
C1—C2—H2	119.8	Se1—C7—H7A	109.5
C2—C3—C4	118.8 (4)	Se1—C7—H7B	109.5
C2—C3—H3	120.6	H7A—C7—H7B	109.5
C4—C3—H3	120.6	Se1—C7—H7C	109.5
C5—C4—C3	121.6 (3)	H7A—C7—H7C	109.5
C5—C4—Br1	119.0 (3)	H7B—C7—H7C	109.5
C3—C4—Br1	119.5 (3)		
			
C7—Se1—C1—C6	−3.0 (3)	C2—C3—C4—Br1	178.8 (3)
C7—Se1—C1—C2	177.8 (3)	C3—C4—C5—C6	0.4 (6)
C6—C1—C2—C3	1.0 (5)	Br1—C4—C5—C6	−178.5 (3)
Se1—C1—C2—C3	−179.8 (3)	C2—C1—C6—C5	−0.7 (5)
C1—C2—C3—C4	−0.6 (5)	Se1—C1—C6—C5	−179.8 (3)
C2—C3—C4—C5	−0.1 (5)	C4—C5—C6—C1	0.0 (5)

### Hydrogen-bond geometry (Å, °)

**Table:**

*D*—H···*A*	*D*—H	H···*A*	*D*···*A*	*D*—H···*A*
C2—H2···C2^i^	0.95	2.84	3.747 (4)	159
C5—H5···C5^ii^	0.95	2.83	3.740 (5)	160

Symmetry codes: (i) *x*−1/2, −*y*+1/2, *z*; (ii) *x*+1/2, −*y*−1/2, *z*.
